# Comparison of Application Value of Different Radiation Dose Evaluation Methods in Evaluating Radiation Dose of Adult Thoracic and Abdominal CT Scan

**DOI:** 10.3389/fsurg.2022.860968

**Published:** 2022-03-25

**Authors:** Jimin He, Guanwei Dong, Yi Deng, Jun He, ZhiGang Xiu, Fanzi Feng

**Affiliations:** ^1^Department of Radiology, The First People's Hospital of Longquanyi District, Chengdu, China; ^2^Department of Rehabilitation, The First People's Hospital of Longquanyi District, Chengdu, China

**Keywords:** volume CT dose index, effective diameter, equivalent diameter of water, body specific dose assessment, CT scanning

## Abstract

**Objective:**

To explore the differences among volumetric CT dose index (CTDI_vol_), body-specific dose assessment (SSDE_ED_) based on effective diameter (ED), and SSDE_WED_ based on water equivalent diameter (WED) in evaluating the radiation dose of adult thoracic and abdominal CT scanning.

**Methods:**

From January 2021 to October 2021, enhanced chest CT scans of 100 patients and enhanced abdomen CT scans of another 100 patients were collected. According to the body mass index (BMI), they can be divided into groups A and D (BMI < 20 kg/m^2^), groups B and E (20 kg/m^2^ ≤ BMI ≤ 24.9 kg/m^2^), and groups C and F (BMI > 24.9 kg/m^2^). The CTDIvol, anteroposterior diameter (AP), and the left and rght diameter (LAT) of all the patients were recorded, and the ED, water equivalent diameter (WED), the conversion factor (*f*_size,ED_), (*f*_size, WED_), SSDE_ED_, and SSDE_WED_ were calculated. The differences were compared between the different groups.

**Results:**

The AP, LAT, ED, and WED of groups B, E, C, and F were higher than those of groups A and D, and those of groups C and F were higher than those of groups B and E (*P* < 0.05). The *f*_size,ED_ and *f*_size, WED_ of groups B, E, C, and F are lower than those of groups A and D, and those of groups C and F are lower than those of groups B and E (*P* < 0.05). CTDI_vol_, SSDE_ED_, and SSDE_WED_ in groups B, E, C, and F are higher than those in groups A and D, and those in groups C and F are higher than those in groups B and E (*p* < 0.05). In the same group, patients with chest- and abdomen-enhanced have higher SSDE_WED_ and SSDE_ED_ than CTDI_vol_, patients with chest-enhanced CT scans have higher SSDE_WED_ than SSDE_ED_, and patients with abdomen-enhanced CT scans have higher SSDE_ED_ than SSDE_WED_ (*P* < 0.05).

**Conclusion:**

CTDIvol and ED-based SSDEED underestimated the radiation dose of the subject exposed, where the patient was actually exposed to a greater dose. However, SSDE_WED_ based on WED considers better the difference in patient size and attenuation characteristics, and can more accurately evaluate the radiation dose received by patients of different sizes during the chest and abdomen CT scan.

## Introduction

With the rapid development of clinical diagnosis and treatment and radiology technology, the application and popularity of CT examination are constantly improving, and the ionizing radiation received by patients is also constantly increasing, which has aroused widespread concern about the potential cancer risk (1). The radiation diagnosed by CT is usually higher than that reported, so it is necessary to accurately evaluate and strictly control the radiation dose of CT. At present, the CT radiation dose index (CTDI_vol_) under the reference standard phantom is usually used to characterize the CT radiation dose clinically, and its value reflects the radiation dose output by the CT equipment but does not consider the patient's body shape factor. In the actual scanning process, different objects have different scanning diameters and attenuation coefficients (2). Therefore, it is inaccurate to evaluate the effective dose of CT in patients with CTDI_vol_ index. The research of Kidoh et al. (3) shows that there is a strong correlation between the specific body dose assessment (SSDE) of patients and the average skin dose, which can more accurately estimate the error of radiation dose reduction. Based on the factors of the patient's body shape, American Medical Physics Association proposed to use effective diameter (ED) and water equivalent diameter (WED) to estimate the specific dose assessment (SSDE) based on the patient's body shape to make up and correct the influence of body shape on CTDI_vol_ and other indicators (4). In this study, we compare the differences among CTDI_vol_, ED-based SSDE_ED_, and WED-based SSDE_WED_ in evaluating the radiation dose of CT scan in the chest and the abdomen of adults with different body mass index (BMI) and discuss the further application of different radiation dose evaluation methods in clinic to provide a reference for clinical research.

## Data and Methods

### General Information

Enhanced chest CT scans of 100 patients and enhanced abdomen CT scans of another 100 patients were collected from January 2021 to October 2021 in our hospital. Inclusion criteria: patients and families members' informed consent; complete clinical image data; clear image, which can meet the research requirements; no metal artifact affecting the radiation dose. Among 200 patients, there were 118 men and 82 women, 21–72 years of age with an average of (48.92 ± 7.24) years, and a body mass index (BMI) of (24.02±3.19) kg/m^2^. This study was approved by the Ethics Committee of our hospital, and the patients and their families provided informed consent.

### Research Methods

GE 128-slice spiral CT scanner was used. The patient was placed in the supine position, feet moved forward, hands raised. The chest scanning was from the top of the lung to the bottom of the lung and abdominal scanning was from the top of the liver to the lower pole of both the kidneys. During the scan, the patient was told to hold his/her breath. The scanning parameters are: adopting automatic tube current modulation technology, the tube current is 80–370 mAs, the tube voltage is 120 kV, the detector collimation is 64 lli.625 mm, the screw pitch is 0.993, and the X-ray tube rotation time is 0.75 s. All scanned images were transmitted to the image storage and transmission system for measurement, and CTDIvol of all patients was recorded. The anteroposterior diameter (AP) and left-right diameter (LAT) of all the patients were measured (at the level of left renal vein trunk and nipple) using workstation measurement software, and ED = $\sqrt{AP\times ALT}$, conversion factor (*f*_size,ED_) = a × e^−*b*×*ED*^, and SSDE_ED_ = f*f*_size,ED_ × CTDI_vol_ were calculated at the same time (5).

An elliptical ROI was selected, including the whole section (except the bed board), the average CT value and area (A) of ROI was recorded, and the WED = $=\sqrt{\left(2\right)}\left(\frac{CT}{1000}+1\right)\times^{\underline{A}},$ the conversion factor (*f*_size, WED_) = a × e^−*b*×*WED*^, and SSDE_WED_ = *f*_size, WED_×CTDI_vol_ for each patient was calculated. In this examination, all subjects used a 16-cm phantom in the scanning except the scout, and the other four enhanced scans used a 32-cm standard phantom to obtain CTDI_vol_ values (6).

A total of 100 patients with enhanced chest CT scanning and 100 patients with enhanced abdomen CT scanning were divided into groups according to BMI. Patients with enhanced chest CT scan were divided into 30 patients in group A (BMI <20 kg/m^2^), 36 patients in group B (20 kg/m^2^ ≤ BMI ≤ 24.9 kg/m^2^), and 34 patients in group C (BMI > 24.9 kg/m^2^). Patients with abdominal enhanced CT scan were divided into 31 patients in group D (BMI <20 kg/m^2^), 35 patients in group E (20 kg/m^2^ ≤ BMI ≤ 24.9 kg/m^2^), and 34 patients in group F (BMI > 24.9 kg/m^2^).

### Statistical Methods

SPSS22.0 software was used for processing, experimental data were measured using mean standard deviation (±s), and one-way analysis of variance was used to compare the differences between the groups in AP, LAT, ED, *f* size_ED_, WED, and *f* size_WED_, respectively. The differences of CTDIvol, SSDE_ED_, and SSDE_WED_ among different BMI groups were compared using the *t-*test. The test level is α=0.05, and the difference is statistically significant when *P* < 0.05.

## Results

### Comparison of AP, LAT, ED, *f*_size,ED_, WED, and *f*_size, WED_ in Patients With Enhanced Chest CT Scan

The AP, LAT, ED, and WED of groups B and C are all higher than those of group A, and that of group C is higher than that of group B, with statistical significance (*P* < 0.05). The *f*_size,ED_ and *f*_size, WED_ of group B and C are lower than that of group A, and that of group C is lower than that of group B (*P* < 0.05), as shown in Table 1.

**Table 1:** Comparison of AP, LAT, ED, *f*_size,ED_, WED and *f*_size, WED_ in patients with enhanced chest CT scan (n,±s).

**Group**	**AP (cm)**	**LAT (cm)**	**ED (cm)**	***f*** **_size,ED_**	**WED (cm)**	***f*** **_size,WED_**
Group A (*n =* 30)	21.63 ± 62.14	30.19 ± 12.51	25.55 ± 51.71	1.44 450.07	20.91 ± 91.45	1.71 790.13
Group B (*n =* 36)	22.65 ± 62.67^a^	32.17 ± 12.73^a^	26.99 ± 91.92^a^	1.37 390.06^a^	22.81 ± 81.52^a^	1.59 580.09^a^
Group C (*n =* 34)	24.99 ± 92.81^a^^b^	35.04 ± 03.05^a^^b^	29.59 ± 52.27^a^^b^	1.25 250.04^a^^b^	26.17 ± 11.96^a^^b^	1.41 410.08^a^^b^
*F-value*	14.496	24.847	34.461	98.750	82.678	72.400
*P-value*	<0.001	<0.001	<0.001	<0.001	<0.001	<0.001

*Compared with group A*,

a *P < 0.05. Compared with group B*,

b *P < 0.05*.

### Comparison of CTDI_vol_, SSDE_ED_, and SSDE_WED_ in Patients With Enhanced Chest CT Scan

The values of CTDI_vol_, SSDE_ED_, and SSDE_WED_ in groups B and C are higher than those in group A, and those in group C are higher than those in group B, with statistical significance (*P* < 0.05). In the same group, SSDE_ED_ and SSDE_WED_ were higher than CTDI_vol_; SSDE_WED_ was higher than SSDE_ED_; and the difference was statistically significant (*p* < 0.05), as shown in Table 2.

**Table 2:** Comparison of CTDI_vol_, SSDE_ED_, and SSDE_WED_ in patients with enhanced chest CT scan (*n*, ±s).

**Group**	**CTDI_**vol**_ (mGy)**	**SSDE_**ED**_ (mGy)**	**SSDE_**WED**_ (mGy)**
Group A (*n =* 30)	3.74 7 0.61	5.32 3 0.73^c^	6.34 3 1.04^cd^
Group B (*n =* 36)	4.36 3 0.67^a^	5.89 8 0.89^a^^c^	6.90 9 1.02^a^^c^^d^
Group C (*n =* 34)	6.53 5 0.75^a^^b^	8.03 0 1.16^a^^b^^c^	9.12 1 1.48^a^^b^^c^^d^
*F-value*	151.948	74.670	50.612
*P-value*	<0.001	<0.001	<0.001

*Compared with group A*,

a *P < 0.05. Compared with group B*,

b *P < 0.05. Compared with CTDIvol in the same group*,

c *P < 0.05. Compared with SSDE_ED_ in the same group*,

d *P < 0.05*.

### Comparison of AP, LAT, ED, *f*_size,ED_, WED, and *f*_size, WED_ in Patients With Abdominal CT Enhanced Scanning

The AP, LAT, ED, and WED of groups E and F are all higher than those of group D, and those of group F are higher than those of group E, with statistical significance (*P* < 0.05). The *f*_size,ED_ and *f*_size, WED_ of group E and F are lower than those of group D, and that of group F is lower than that of group E, with statistical significance (*P* < 0.05), as shown in Table 3.

**Table 3:** Comparison of AP, LAT, ED, *f*_size,ED_, WED, and *f*_size, WED_ in patients with abdominal CT enhanced scanning (n, ±s).

**Group**	**AP (cm)**	**LAT (cm)**	**ED (cm)**	***f*** **_size,ED_**	**WED (cm)**	***f*** **_size,WED_**
Group D (*n =* 31)	18.92 ± 91.84	27.84 ± 82.01	22.95 ± 91.24	1.59 590.17	23.82 ± 82.06	1.54 580.18
Group E (*n =* 35)	20.16 ± 11.97^a^	29.23 ± 2.27^a^	24.28 ± 21.37^a^	1.51 520.15^a^	25.68 ± 62.21^a^	1.44 460.16^a^
Group F (*n =* 34)	21.75 ± 72.03^a^^b^	31.49 ± 42.64^a^^b^	26.17 ± 11.52^a^^b^	1.41 410.13^a^^b^	28.94 ± 92.74^a^^b^	1.27 290.13^a^^b^
*F-value*	17.223	20.469	211.461	64.629	39.462	22.746
*P-value*	<0.001	<0.001	<0.001	<0.001	<0.001	<0.001

*Compared with group D*,

a *P < 0.05. Compared with group E*,

b *P < 0.05*.

### Comparison of CTDI_vol_, SSDE_ED_, and SSDE_WED_ in Patients With Abdominal CT Enhanced Scanning

The values of CTDI_vol_, SSDE_ED_, and SSDE_WED_ in groups E and F are higher than those in group D, and those in group F are higher than those in group E, with statistical significance (*p* < 0.05). In the same group, SSDE_ED_ and SSDE_WED_ were higher than CTDI_vol_, SSDE_ED_ was higher than SSDE_WED_, and the difference was statistically significant (*P* < 0.05), as shown in Table 4.

**Table 4:** Comparison of CTDI_vol_, SSDE_ED_ and SSDE_WED_ in patients with abdominal CT enhanced scan (*n*, ±s).

**Group**	**CTDI_**vol**_ (mGy)**	**SSDE_**ED**_ (mGy)**	**SSDE_**WED**_ (mGy)**
Group D (*n =* 31)	3.52 5 0.55	5.52 5 0.91^c^	5.35 3 0.84^c^^d^
Group E (*n =* 35)	4.19 1 0.58^a^	6.28 2 0.96^a^^c^	5.96 9 0.89^a^^c^^d^
Group F (*n =* 34)	6.09 0 0.69^a^^b^	8.50 5 1.17^a^^b^^c^	7.67 6 1.02^a^^b^^c^^d^
*F-value*	155.919	39.510	56.389
*P-value*	<0.001	<0.001	<0.001

*Compared with group D*,

a *P < 0.05. Compared with group E*,

b *P < 0.05. Compared with CTDIvol in the same group*,

c *P < 0.05. Compared with SSDE_ED_ in the same group*,

d *P < 0.05*.

## Discussion

The area on the parallel lines along the axis (*z*) under the single-layer scanning dose distribution curve is denoted by CDTI. Due to some limitations in its measurement, CDTI_100_, CDTI_W_, and CTDI_vol_ were subsequently exported. CTDI_vol_ can be used to compare the radiation doses from different CT scanners. CTDI_vol_ represents the radiation dose value of one-layer images along the rotation axis, which is the radiation dose output level calculated based on the standard phantom. However, it has nothing to do with the scanning length. It reflects the radiation dose level output of the equipment, rather than the radiation dose received by patients, and can not truly reflect the radiation dose assessment received by patients with different body types (7, 8). Therefore, when CTDI_vol_ is used to evaluate the radiation dose received by patients, the problem of underestimating the radiation dose received by patients with low body weight will appear (9). CTDI_vol_ is very sensitive to the changes of scanning parameters, such as tube voltage, tube current, X-ray tube rotation time, etc. For different human bodies, its scanning diameter is different and the radiation dose is different. The emergence of SSDE parameter solves this problem.

Body-specific dose estimation is a CT dose estimation value corrected by the patient's body shape. It is obtained by standardizing CTDI_vol_ with *f* on the basis of CTDI_vol_. Considering *f* , a factor related to the patient's body shape, it can more accurately evaluate the actual radiation dose received by the patient (10). Australia, New Zealand, and other countries have suggested using SSDE in chest examination to establish the dose reference (11, 12). The results show that with the increase of BMI, AP, LAT, ED, and WED of different types of patients' chest and abdomen enhanced CT scans all increased to varying degrees, while *f*_size,ED_, *f*_size, WED_ showed a downward trend. In this study, the standard phantom with a diameter of 32 cm was used, but the ED of abdominal CT scan in most patients was <30 cm, and only 6 patients had an ED that fluctuated in the range of 30–32 cm, which was obviously different from that of the standard phantom. This study also shows that CTDI_vol_ is used to evaluate the radiation dose in enhanced CT scans of the chest and abdomen, which is obviously lower than that of SSDE_ED_ and SSDE_WED_, and there is a problem of underestimating the actual radiation dose.

The SSDE effectively makes up for the deficiency of CTDI_vol_ in body shape difference and tissue attenuation. Based on the method of ED evaluation, it is assumed that the patient's body cross-section is elliptical, and the internal components are all water, and then the circle diameter F equal to the elliptical area is used to correct. However, it is not suitable for this changeable and irregular geometric shape of the human body, and the radiation dose will be underestimated when the tissue density of the CT scan is quite different from that of water (13, 14). The method of calculating radiation dose based on WED can consider the size and X-ray attenuation factors of different parts of the patient's chest and abdomen. It is closely related to X-ray imaging and is suitable for irregular and uneven tissues of the human body (15, 16). However, there are few SSDE based on WED. This study discusses the application of SSDE based on WED calculation in the adult thorax and abdomen by comparing the differences of three different body-specific dose assessments in the body.

The results show that with the increase of BMI, the values of SSDE_ED_ and SSDE_WED_ gradually increase. The SSDE_WED_ of patients with enhanced CT scan in the chest is higher than that of SSDE_ED_, while that of patients with enhanced CT scan in the abdomen is lower than that of SSDE_ED_. Since the air content in the chest is obviously lower than that in the water model and the overall attenuation in the chest area is obviously lower than that in the water model, the average CT value corresponding to X-ray attenuation *in vivo* is negative, while the density of abdomen tissues is roughly the same as that in the water model, the overall attenuation is consistent with that in the water model, and the average CT value corresponding to X-ray attenuation is positive. With the increase of BMI, the difference between SSDE_ED_ and SSDE_WED_ also gradually increases (17).

There are still some limitations in this research. This study involves only adult patients. Some studies based on ED SSDE_ED_ show that it is meaningful for infants and young children (18). At the same time, the number of cases distributed in different BMI ranges is relatively small, which needs to be further discussed in the follow-up study.

To sum up, CTDIvol and ED-based SSDEED underestimated the radiation dose to which the subject was exposed, and the patient was actually exposed to a greater dose. However, SSDE_WED_ based on WED better considers the difference in patient size and attenuation characteristics, and can more accurately evaluate the radiation dose received by patients of different sizes during the chest and abdomen CT scan.

## Data Availability Statement

The original contributions presented in the study are included in the article/supplementary material, further inquiries can be directed to the corresponding author/s.

## Ethics Statement

This study was approved by the Medical Ethics Committee of the First People's Hospital of Longquanyi District. The patients/participants provided their written informed consent to participate in this study. Written informed consent was obtained from the individual(s) for the publication of any potentially identifiable images or data included in this article.

## Author Contributions

JiH is responsible for the writing of the paper. GD is responsible for the design of the study. YD is responsible for the inclusion of cases. JuH is responsible for the evaluation of the results. ZX is responsible for the statistics of the data. FF is responsible for the guidance of the entire study. All authors contributed to the article and approved the submitted version.

## Conflict of Interest

The authors declare that the research was conducted in the absence of any commercial or financial relationships that could be construed as a potential conflict of interest.

## Publisher's Note

All claims expressed in this article are solely those of the authors and do not necessarily represent those of their affiliated organizations, or those of the publisher, the editors and the reviewers. Any product that may be evaluated in this article, or claim that may be made by its manufacturer, is not guaranteed or endorsed by the publisher.
